# Mutations in the SARS-CoV-2 spike protein modulate the virus affinity to the human ACE2 receptor, an *in silico* analysis

**DOI:** 10.17179/excli2021-3471

**Published:** 2021-03-08

**Authors:** Joseph Thomas Ortega, Flor Helene Pujol, Beata Jastrzebska, Hector R. Rangel

**Affiliations:** 1 Department of Pharmacology, Cleveland Center for Membrane and Structural Biology, School of Medicine, Case Western Reserve University, Cleveland, OH, USA; 2 Laboratorio de Virología Molecular, Centro de Microbiología y Biología Celular, Instituto Venezolano de Investigaciones Científicas, Apdo 20632, Caracas 1020A, Venezuela

**Keywords:** B.1.1.7, COVID-19, SARS-CoV-2, spike, variants

## Abstract

The increasing number of SARS-CoV-2 variants associated with highly transmissible phenotypes is a health-public concern in the current pandemic scenario. Herein, we developed a comprehensive *in silico* analysis of the changes occurring upon mutations in the viral spike. We focused on mutants located in the receptor-binding domain of the viral spike protein and analyzed whether these mutants modulate the interaction with the human host receptor angiotensin-converting enzyme II (ACE2). Thirty-two highly prevalent mutants were retrieved from the GISAID database, and their structural models were built using the SWISS-Model server. The stabilization effect for each mutation was assessed by the DUET and DeepDGG software. By applying molecular docking using both Z-Dock and Haddock software we found that multiple mutations, including A475V, V455E, V445L, and V445I, resulted in the higher binding free energy as compared to the wild type (WT) spike protein, thus had a destabilizing effect on the binding to ACE2. On the other hand, several mutants, including the most prevalent N501Y and B.1.1.7 variants, as well as the K444R, L455F, Q493R, and Y505W variants exhibited lower binding free energy as compared to the WT spike. These mutants showed an increased number of electrostatic interactions with ACE2 than the WT spike protein, and they changed the interaction pattern of the neighboring residues. Together, the results presented in this study contribute to a better understanding of the changes in the interaction between SARS-CoV-2 and the human host ACE2 receptor associated with point mutations in the viral spike protein.

## Introduction

Viruses from the Coronaviridae family such as Severe Acute Respiratory Syndrome Coronavirus (SARS-CoV) and Middle Eastern Respiratory Syndrome Coronavirus (MERS-CoV) have been related to important epidemiological outbreaks (Sharma et al., 2020[27]). Yet, another betacoronavirus, SARS-CoV-2, and its associated disease named COVID-19 have rapidly expanded worldwide becoming the third pandemic related to coronavirus in the first 20 years of the 21^st^ century. Coronaviruses are enveloped viruses with a ~ 32 kb positive-sense single-strand RNA. The viral evolution had converged in a highly regulated replication cycle to maintain the genetic stability of their long RNA genome (Romano et al., 2020[26]). Coronavirus has the ability to proofreading processing in their replication complex, thus, the presence of mutations is lower in comparison to other RNA viruses (Romano et al., 2020[26]). However, the accumulation of viral variants related to genomic mutations still occurs in coronavirus. These viral variants play a pivotal role in viral escape to selective pressure such as *e.g.,* host immunological system, drugs, or vaccines. Also, in SARS-CoV-2, changes in the host range, virulence, and tropism are driven mainly by the selection of viral variants through mutations (Romano et al., 2020[26]). The viral spike is responsible for the recognition of the host receptor angiotensin-converting enzyme II (ACE2). The changes that already occurred in the viral spike of SARS-CoV-2 likely increased the efficiency of the virus-ACE2 receptor interactions in comparison to SARS-CoV (Ortega et al., 2020[18]). Although the infection with SARS-CoV-2 in humans is a recent evolutionary event, the number of mutations in the gene encoding spike protein is increasing continuously (Bobay et al., 2020[2], Sironi et al., 2020[29]). The advances in genome deep sequencing and its analysis increased the epidemiological vigilance in naturally occurring variants of the virus. In the case of SARS-CoV-2, epidemiological reports show a high diversity of mutants in the viral spike protein. Some variants have been associated with increased stability of virions and consequent high transmissibility, *e.g*. 1) The D614G mutation in the spike protein was first detected in Europe in the early phase of pandemic, and currently it is widely spread around the globe. This mutation was found at low prevalence before March 2020, however, in June 2020 the occurrence of the D614G variant was reported in over 70 % of the SARS-CoV-2 published sequences (Bobay et al., 2020[2]; Groves et al., 2021[9]; Korber et al., 2020[11]); 2) The B.1.1.7 linage that first appeared in the United Kingdom became rapidly worldwide distributed, reaching the United States in January 2021. This linage has a large number of genetic changes, especially in the receptor-binding domain (RBD), including deletion of residues 69 and 70 and a non-synonymous substitution of N to Y at residue 501, one of the key contact residues with the ACE2 host receptor that could be related to its increased virulence. This linage also exhibited a change at the residue 681 (P681H), one of the four residues comprising the furin cleavage site located between S1 and S2 domains in the spike protein (Rambaut et al., 2020[24]). An increase in the number of positively charged residues in this region in SARS-CoV-2 has already improved the virus interaction with the furin cleavage site as compared to SARS-CoV (Hoffmann et al., 2020[10]). Thus, P681H residue substitution could additionally enhance this interaction. Nevertheless, the frequency of these viral variants increased locally and globally during the current outbreak, suggesting that these changes improved viral fitness.

To better understand the dynamics of the SARS-CoV-2 pandemic, close tracking of the changes occurring in the viral spike is required. These mutations could affect the binding affinity to the human host receptor or result in the generation of variants with decreased neutralization effect of antibodies by changing the antibody recognition site. Thus, to learn about the mutation-related changes in the SARS-CoV-2 fitness, in this study, we performed an *in silico* analysis of 32 mutants reported with high frequency in clinical isolates around the world. We assessed the affinities of these variants to the main human host receptor ACE2 in comparison to WT SARS-CoV-2 and examined whether these mutants alter or improve the interaction between the spike protein and the ACE2 receptor.

## Materials and Methods

### Viral variants data and mutation stability analysis 

The viral variants data was retrieved from GISAID and CoV-Glue webservers, on December 27, 2020 (Elbe and Buckland-Merrett, 2017[6]; Singer et al., 2020[28]). Each mutation was individually evaluated by using DUET (Pires et al., 2014[23]) and DeepDDG (Cao et al., 2019[3]) online software that uses neural networks to calculate the thermodynamic changes to predict the stability of the point mutation. The ΔΔG values expressed in kcal/mol were tabulated individually and the prediction for each mutation in terms of its stabilizing or destabilizing effect was analyzed following the user guide. 

### Protein modeling

The sequence for the SARS-CoV-2 viral spike protein was retrieved from the Uniprot server (sequence number P0DTC2) and homology structural models were built by using the tools of the SWISS-MODEL modeling server and the DeepView/Swiss-PdbViewer 4.01 software (Arnold et al., 2006[1]). For each mutant, the quality of each structure was validated via ProSA-web and PROCHECK programs (Laskowski et al., 1993[13]; Wiederstein and Sippl, 2007[39]). Hydrogen atoms were added, and partial charges were assigned for the energy refinement. The obtained models were subjected to molecular dynamic (MD) simulations using NAMD 2.12 (Phillips et al., 2005[21]), as described in Ortega et al. (2020[17], 2019[19]) using the CHARMM force field and Gasteiger charges (Vanommeslaeghe et al., 2010[33]). The obtained structures represent the lowest energy frame of the MD simulations. 

### Docking

The crystal structure of the SARS-CoV-2 spike protein bound to the human ACE2 receptor (PDB code: 6M0J) and the structure of the human ACE2 receptor (PDB code: 1R42) were downloaded from the Protein Data Bank. Each mutant and the WT spike protein obtained from the 6M0J structure were evaluated. The protein preparation was carried out as described in the previous paragraph. Then, the binding patterns and affinity estimations for the interaction between the viral spike protein and the ACE2 receptor were performed using molecular docking. Two programs Z-Dock (Pierce et al., 2014[22]) and PROGIDY (Xue et al., 2016[41]) were used to obtain the docking complexes. First, the docking between a ligand (RBD of the WT or mutant spike protein) and a receptor (ACE2) was performed with Z-Dock software. Then, the obtained complexes were processed and analyzed by using the tools of PRODIGY software. Furthermore, docking analysis for each mutant of the spike protein was assayed using the Haddock server (van Zundert et al., 2016[32]). The results obtained with each software were clustered and analyzed considering the free binding energies and the main interacting residues in each spike protein-ACE2 receptor complex.

### Molecular dynamics

We used two software to carry out MD simulation for the selected spike protein mutants, NAMD on VegaZZ (van Zundert et al., 2016[32]) and CABS-flex software (Kuriata et al., 2018[12]). For VegaZZ, the calculations were performed with the NAMD software using the CHARMM force field parameters. The PDB files for each mutant were prepared for MD analyses and the followed structure refinements using fragment-guided molecular dynamics and the FG-MD algorithm available on the Zhang laboratory webpage (Zhang et al., 2011[42]). The structures were embedded into a solvation water box, followed by ionization and neutralization of simulation with Na ions. MD simulations were performed with coupled temperature (300° K) and pressure (1 bar) for 10 ns. The complex for each structure was minimized for 10,000 conjugate gradient steps. Alternatively, the PDB files were submitted to the CABS-flex server to further assess the stability of the spike protein mutants-ACE2 complexes, and the parameters were adjusted as default. The MD simulations output data obtained with both software were analyzed according to root-mean-square deviation (RMSD).

## Results

### Mutation stability

**Figure 1:**
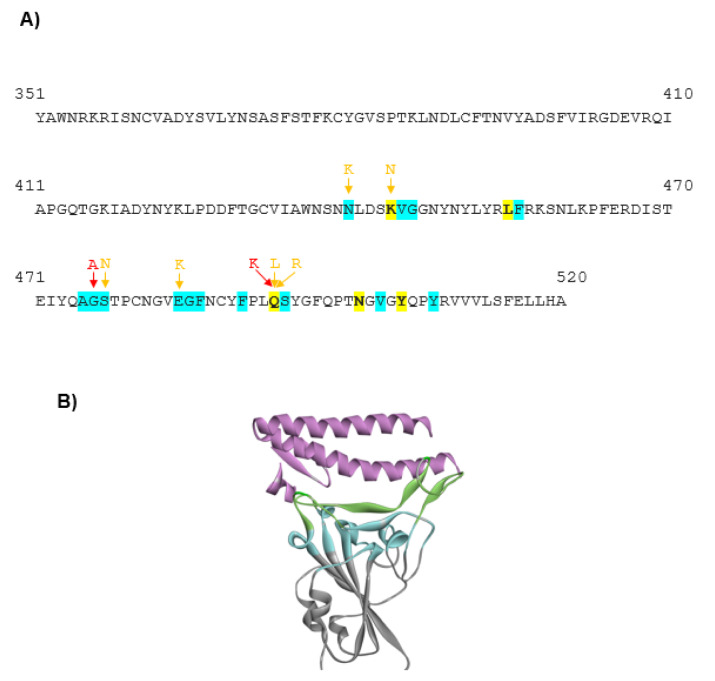
The SARS-CoV-2 spike protein. A) The sequence of the SARS-CoV-2 spike protein RBD (residues 330-510) is shown. The mutations evaluated in this work are indicated. The stabilizing mutations predicted by both DUET and DeepDDG software are colored in red, while mutations predicted by a single software are colored in yellow. B) The structure of the SARS-CoV-2 spike protein RBD in the complex with ACE2 receptor (PDB code: 6M0J). The viral spike protein is shown in gray. However, the region containing the residues directly interacting with ACE2 is indicated in green. The region containing the residues that do not interact directly with ACE2 but their substitution could modulate the spatial organization of the directly interacting residues is indicated in blue. The human host receptor ACE2 is shown in pink.

The mutations in the RBD of the viral spike protein led to an increase in the prevalence of the certain SARS-CoV-2 variants that could spread faster than the WT virus. These mutants actively circulate in the human population worldwide and may exhibit different sensibility to neutralization by monoclonal antibodies (Weisblum et al., 2020[38]). Thus, it is important to understand, which of these mutants have the most harmful effects on the human population. An increase in the binding stability of the virus to the host receptor could be one of the reasons for the higher virus infectivity. Thus, in this work, we analyzed 32 SARS-CoV-2 mutants with high occurrence available in the GISAID database and the CoV-GLUE server. The number of the reported sequences containing these mutations is indicated in Table 1(Tab. 1). For each mutant, we generated the models using the SWISS-MODEL server. The models showing the best match with the SARS-CoV-2 viral spike (PDB code 7DK6 or 7C01) were selected for further analyses. The sequence of the SARS-CoV-2 spike protein RBD, indicating the amino acid substitutions that cause the changes in the host selectivity, is shown in Figure 1A(Fig. 1). The region containing the residues involved in the interaction with ACE2 is indicated in the 3D structural model of the spike protein in Figure 1B(Fig. 1). To gain more insight into the changes within the structural microenvironment of the spike protein RBD caused by the mutations, first, we analyzed the Miyata distances associated with the physico-chemical properties (polarity, size, among others) of each substituted residue. The value of 0.06 indicates the most similar residue pair, such as Ala and Pro, while the value of 5.13 indicates the most dissimilar residue pair, such as Gly and Trp. In our analysis the highest Miyata values, indicating the largest differences between the WT and spike protein mutants were found for the following residue substitutions: N501Y, F490S, Q493L, G446V, and Y508H. 

Next, to evaluate the changes in the spike-ACE2 complex stability associated with the mutations, we employed an integrated computational approach for studying missense mutations in proteins using two web servers, DUET and DeepDDG, which consolidate two complementary approaches, obtained by machine learning algorithms. The ΔΔG change induced by each mutation and the prediction of its effect on the binding stability of the spike protein to ACE2 are shown in Table 2(Tab. 2). The substitutions G476A, K444N, N440K, Q493K, Q493L, and Q493R were identified as stabilizing by the DUET software. The stabilization effect of the G476A and Q493K mutations was confirmed by the DeepDDG software. The other analyzed mutants were assigned as destabilizing by both the DUET and DeepDDG software, indicating that the stabilization prediction by each software is highly correlative in our experimental conditions.

### Interaction between the viral mutants and the human ACE2

**Figure 2:**
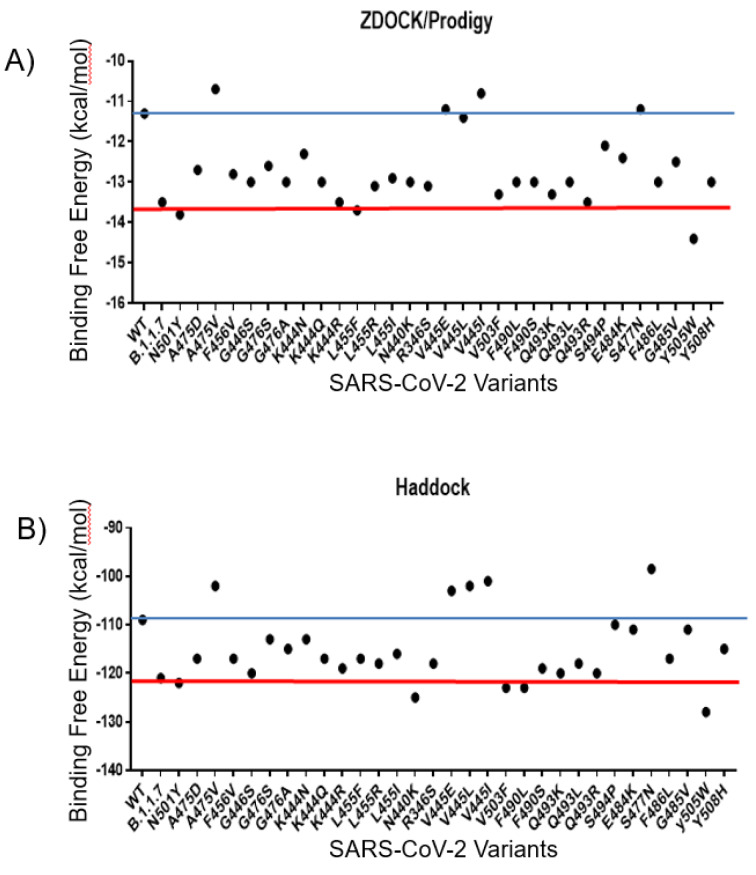
The molecular docking analysis of the SARS-CoV-2 mutants. A) The molecular docking results were obtained with the Z-Dock server and B) with the Haddock server. The red line denotes the mutants with similar values to the N501Y mutation and the blue line denotes the values for the mutants with similar values to the WT spike protein.

The ACE2 receptor is the main receptor related to the viral entry of SARS-CoV-2 in the human host. Thus, the comparison of the interactions between ACE2 and WT spike or the mutants could result in obtaining clues about the changes in the binding affinity of these mutants. To generate the spike protein mutants and ACE2 complexes, we first used the Z-Dock software followed by further interaction analysis with PRODIGY software. The WT spike protein interacted with the ACE2 receptor with the binding free energy of -11.8 kcal/mol. Several mutants, including A475V, V455E, V445L, and V445I interacted with the higher binding free energy of -10.7 kcal/mol, -11.2 kcal/mol, -11.4 kcal/mol, and -10.8 kcal/mol, respectively, resulting in less stable interaction with ACE2 than the WT spike protein. The other investigated mutants, including the B.1.1.7 (-13.4 kcal/mol), K444R (-13.8 kcal/mol), L455F (-13.7 kcal/mol), Q493R (-13.5 kcal/mol) and Y505W (-14.4 kcal/mol) had the binding free energy lower than the WT spike protein, which indicated their higher affinity for the human ACE2 receptor. Interestingly, the Y505W mutant showed the lowest binding free energy among the analyzed mutants. To validate the results obtained with the Z-Dock/PRODIGY servers, an additional molecular docking analysis was performed for each spike protein-ACE2 complex using the Haddock server that is broadly used to study protein-protein docking and uses an algorithm developed based on different parameters than the Z-Dock server. The results obtained with using the Haddock server showed a similar trend as the results obtained with the Z-Dock server. The high scores (low binding affinity), as well as the low scores (high binding affinity), were assigned to the same variants by both servers. The summary of the molecular docking results obtained with both Z-Dock and Haddock servers is shown in Figure 2(Fig. 2). 

**Figure 3:**
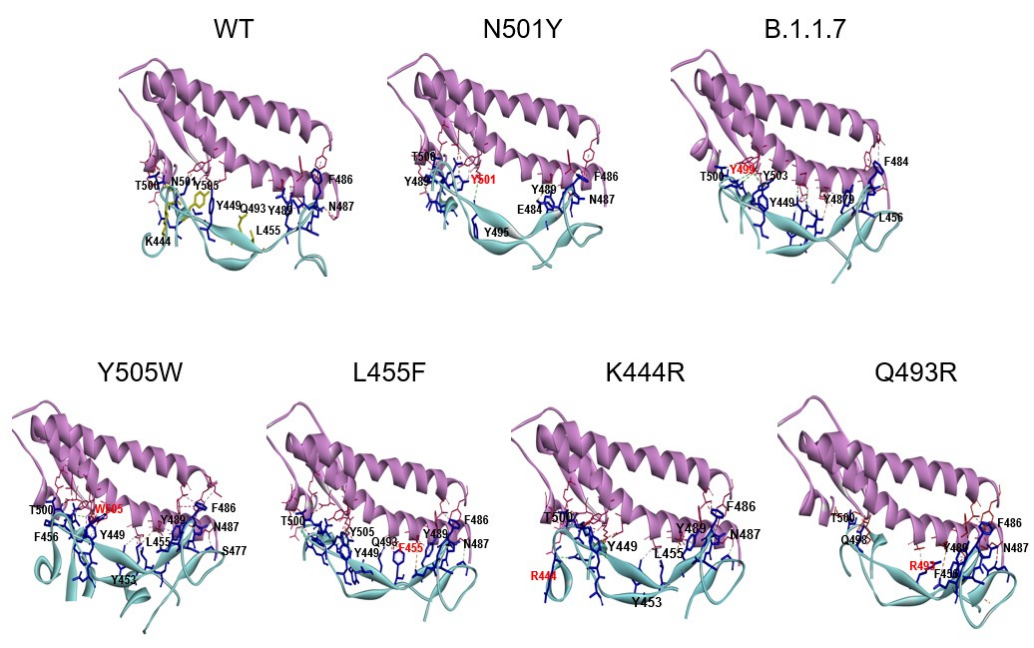
The binding pattern of the SARS-CoV-2 mutants to the ACE2 receptor. A close up view for the complex obtained by the molecular docking analysis for the spike protein mutants with the binding free energies similar to the N501Y mutant. The WT spike-ACE2 complex is also included. The viral spike protein and the receptor-interacting residues are shown in blue and the human host ACE2 receptor is shown in pink.

The N501Y spike protein mutant was described as a highly transmissible variant in the current SARS-CoV-2 pandemic (Leung et al., 2021[14]). Our molecular docking analysis revealed the binding free energy for the N501Y mutant to be -13.8 kcal/mol. Thus, our further analysis was focused on the mutants that exhibited similar or lower binding free energy than that found for the N501Y mutant, including the B.1.1.7, K444R, L455F, Q493R, and Y505W variants (see Table 3(Tab. 3)). The binding patterns with the human ACE2 for each variant are shown in Figure 3(Fig. 3). In addition, the number and the characteristics of these mutants-ACE2 interactions are shown in Table 3(Tab. 3). The mutants with the lower binding free energy produced a higher number of interactions with the ACE2 receptor as compared to the WT spike protein. As we found, for the N501Y mutant, and variant B.1.1.7, which also contains the N501Y substitution, the Tyr residues produced 9 and 7 interactions with the ACE2 receptor, respectively in comparison to the Asp residue present in the WT spike protein that produced only 4 interactions. In the case of the Y505W mutant, exhibiting the lowest binding free energy, the number of direct interactions between the Trp residue and ACE2 increased to 8 as compared to the Tyr residue in the WT spike protein that produced 5 interactions. Additionally, in this particular mutant, the number of indirect interactions with the surrounding residues increased to 15, while only 5 were found in the WT spike protein. Furthermore, each of these mutations caused a change in the pattern and type of interactions produced in the RDB region. Similar structural effects were observed for the L455P and K493R mutants, which also produced changes in the number and type of interactions. In the WT spike protein, the residue K444 does not interact directly with the ACE2 receptor. Similarly, to the WT spike protein, the K444R mutant did not show any direct interactions with ACE2. However, the structural rearrangement associated with this amino acid substitution resulted in an increased number of interactions with the residues proximal to the mutant, including residues N437, N439, S443, and G446 that were not present in the WT spike protein. Altogether, these changes in the number and type of interaction could explain the decrease in the binding free energy values and a consequent increase in the binding affinity of SARS-CoV2 to the human host ACE2 receptor. A detailed list of the interactions identified for these spike protein mutants is shown in Table 4(Tab. 4). 

To validate further the results obtained by the molecular docking, the selected spike protein mutants (N501Y and B.1.1.7) were analyzed by the MD simulations and compared to the WT spike protein. First, the spike protein-ACE2 receptor complexes were evaluated using the CABS-flex software. This software enables an efficient modeling procedure for short simulations being able to produce an analysis of the protein dynamics consistent with the dynamics obtained from 10-nanoseconds MD simulations with the most popular force-fields. Next, the dynamics of these complexes were evaluated using NAMD and the CHARMM force field on VegaZZ software. The results with both approaches showed a similar pattern in the flexibility of the analyzed structures, expressed as RMSD, as compared to the parental structures obtained from the molecular docking analyses. These results obtained for the main residues located in the RBD are shown in Supplementary Figure 1. The median average RMSDs obtained with the CASB-flex software were 1.05±0.77 Å for WT, 0.99±0.65 Å for the N501Y mutant, and 0.91± 0.58 Å for the B.1.1.7 mutant. For the VegaZZ software, the average RMSDs were 2.33±0.55 Å for WT, 2.45±0.54 Å for the N501Y mutant, and 2.37±0.32 Å for the B.1.1.7 mutant. Despite different algorithms used by both software, the results showed a similar trend of change in the residue flexibility in the SARS-CoV-2 spike protein mutants as compared to the WT protein, further validating our docking results.

## Discussion

The spike protein of SARS-CoV-2 is responsible for initiating the interaction between the virus and the host receptor ACE2 in human cells. The mutations in this protein most likely would lead to conformational changes that could modulate the viral infectivity (Ortega et al., 2020[18]; V'kovski et al., 2021[35]). Multiple factors affect viral diversity and the consequent viral fitness. However, in the SARS-CoV-2 pandemic, the replication errors introduced by the RNA-dependent RNA polymerase and the size of the human population among which the virus circulates are the most critical for the observed increase of viral variants (Pachetti et al., 2020[20]). The viral spike protein is also a major target for the therapeutic monoclonal antibodies and antibodies generated by the human host defense system either upon infection or after vaccination (Lip et al., 2006[15]). The binding of such neutralizing antibodies to the viral spike protein may block the virus's ability to infect new cells. However, mutations occurring in the viral genome could result in emerging of so-called 'escape variants' not susceptible to neutralization by these antibodies. This phenomenon is well described for viruses such as HIV, HCV, and influenza among others (Doud et al., 2017[5]; Wei et al., 2003[37]). However, less is known whether SARS-CoV-2 variants have changed their ability to bind the human ACE2 receptor and if they could evade the antibodies recognizing WT SARS-CoV-2. To gain insight on how mutations in SARS-CoV-2 change the virus affinity to ACE2, we analyzed 32 of the most prevalent mutants that occurred in the receptor-binding domain of the SARS-CoV-2 spike protein. An amino acid replacement can change the physico-chemical distance resulting in the structural change that could either stabilize or destabilize the ligand-receptor complex. Most of the mutations in the SARS-CoV-2 spike protein analyzed in this study produced destabilizing effects on the spike. However, these mutations were located primarily in the protein loops, which due to their high flexibility could likely tolerate the conformational changes associated with the residue substitution. Nevertheless, 8 mutations resulted in the enhanced spike stability. In terms of interaction with the ACE2 receptor, the analyzed SARS-CoV-2 spike protein mutants showed a wide range of binding affinity. Most mutants exhibited lower binding free energy, thus higher binding affinity to the host receptor. We focused on the mutants, which showed enhanced binding affinities to ACE2. In the most prevalent mutants such as N501Y and B.1.1.7, the substitution of Asp to Tyr resulted in a substantial conformational reorganization that led to an increased number of interactions and changed nature of these interactions between the spike protein and ACE2 receptor. Specifically, an increase in the number of hydrogen bonds and pi-pi interactions as well as the change from a charged to an aromatic residue with an OH group as a strong donor-acceptor for the hydrogen bond formation could explain the increased affinity of these viral variants for the host receptor. The highest increase in the receptor binding affinity was obtained for Y505W. Although, in this case, both residues are aromatic, the volume occupied by Trp is larger than the one of Tyr. Thus, residue substitution in the Y505W mutant likely introduced the conformational rearrangements that allowed the surrounding residues to interact better with the ACE2 receptor. In addition, due to the presence of two aromatic rings, Trp produced more pi-pi interactions than Tyr. Although currently, the Y505W variant is not found frequently, it deserves further analysis for genomic surveillance over time as this mutation significantly increases the binding affinity to human ACE2. The binding free energy of this mutant to ACE2 is lower than of the WT spike protein and both currently most prevalent variants, N501Y and B.1.1.7. In addition, our results are in concordance with other reports indicating that the S477N mutation did not produce changes in the secondary structure of the SARS-CoV-2 spike protein. Whereas the mutation of N501Y resulted in the conformational rearrangements increasing its affinity to the human ACE2 receptor in comparison to the S477N variant (Mathavan, 2020[16]).

The above-mentioned mutations are located in the binding motif of the RBD that directly interacts with the host receptor ACE2 (Ortega et al., 2020[18]). However, as we found for some of the mutants, the substitution in the residues that are not directly involved in the interaction with the receptor could also enhance the binding affinity of the spike protein to ACE2, likely through the conformational changes that improve the spike protein accommodation within the receptor. Thus, a cooperative or an indirect effect could be proposed as an alternative to explain an increased binding affinity of the mutants that occurred in a different location than the residues directly interacting with the receptor. A similar explanation has also been proposed by other authors for the S494P spike protein variant (Chakraborty, 2021[4]). 

In this study, we focused our analysis on single mutants and only one variant B.1.1.7 associated with multiple genetic changes, including a change in the RBD and the furin cleavage site. However, the selection of double or multiple residue substitutions in the same genomic region occurring at the same time has also been reported. A similar phenomenon has been reported for other viruses such as HIV and HCV under treatment with antivirals (Vega et al., 2004[34]). Thus, in order to fight the current pandemic keeping track of the changes constantly occurring in the SARS-CoV-2 viral population, epidemiological vigilance, the correct reports, and availability of the data are required. Some of the SARS-CoV-2 variants that are already circulating in the human population gained the ability to evade *in vitro* the monoclonal antibodies recognizing the spike protein (Weisblum et al., 2020[38]). However, the viral spike protein variants with higher affinity to the human host ACE2 receptor analyzed in this study did not correlate with the mutants exhibiting low susceptibility to monoclonal antibodies *in vitro*. Additionally, as recently reported, the host antibodies generated after vaccination with the currently available mRNA vaccine could neutralize the N501Y variant (Xie et al., 2021[40]). This is related to a different location (residues 301 to 430) of the antibody recognition site within the S1 region of the spike protein than the main receptor-binding motif (Ewer et al., 2021[7]; Ravichandran et al., 2020[25]). It has been reported recently that the E484K mutant has a lower affinity to the neutralizing antibodies than the WT spike (Greaney et al., 2020[8]). This mutant is located in the RBD but did not improve the interaction with the ACE2, showing the binding affinity similar to WT. Thus, changes in the virus affinity to the host receptor do not have to correlate with a decrease in the susceptibility to spike recognizing antibodies (Weisblum et al., 2020[38]; Xie et al., 2021[40]). Recently, the scientific and health community also has focused on the new variants called the South African (B.1.351) and Brazilian (P.1) variants that carry several mutations including both the N501Y and E484K (Tang et al., 2021[30]; Toovey et al., 2021[31]). Thus, their high transmissibility is most likely related to increased binding affinity to the human host receptor through the N501Y mutation and reduced response to antibodies associated with the E484K mutation. Indeed, the B.1.351 variant shows a dramatic ~10-fold resistance to neutralization by antibodies administrated therapeutically or antibody generated after vaccination with the currently available vaccines (Wang et al., 2021[36]). Thus, under the present pandemic scenario with a high rate of new viral variants appearing, the probability of the selection of other mutants with an increased viral fitness is high. In order to prevent the selection and spread of such new viral variants a boost in the vaccination efforts is required to make it largely available to the general human population. Additionally, modification of the vaccine formulation might be needed to warrant the production of antibodies neutralizing the South African and Brazilian variants that are resistant to the currently available vaccines. 

Altogether, the results presented in this study contribute to a better understanding of whether the changes in the viral spike associated with point mutations modulate the interaction with the human host ACE2 receptor. However, further analyses employing computational, structural, and biochemical approaches are required to fully delineate the structural details of the specific mutations triggering the higher SARS-CoV-2 virus transmissibility.

## Notes

Beata Jastrzebska and Hector R. Rangel (Laboratorio de Virología Molecular, Centro de Microbiología y Biología Celular, Instituto Venezolano de Investigaciones Científicas, Caracas, Venezuela; Phone: 58-412-7075300, E-mail: hrangel2006@gmail.com) contributed equally as corresponding authors.

## Supplementary Material

Supplementary material

## Figures and Tables

**Table 1:**
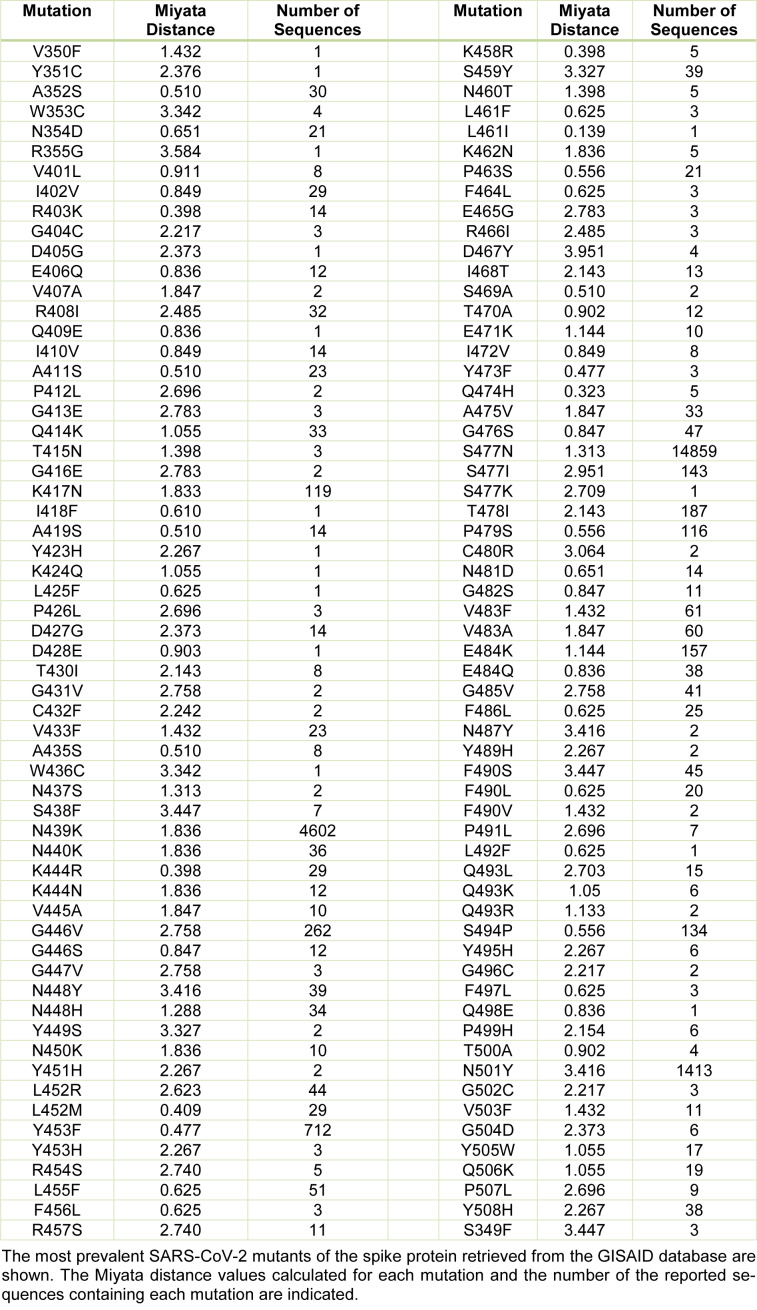
The changes in the structural microenvironment of the SARS-CoV-2 spike protein related to the amino acid substitution

**Table 2:**
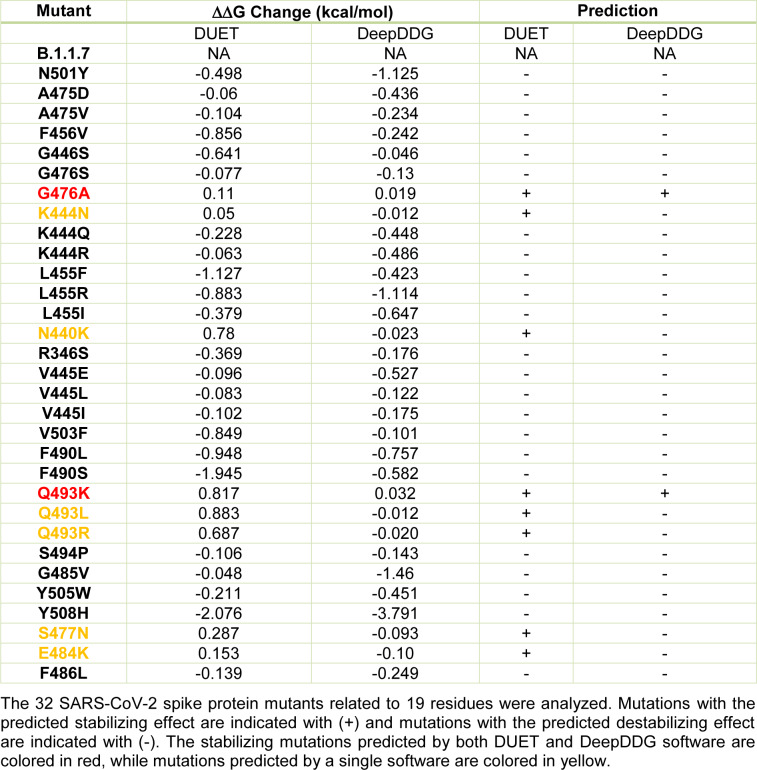
The effect of amino acid substitution in the RBD of the SARS-CoV-2 spike protein on the stability of the spike microenvironment

**Table 3:**
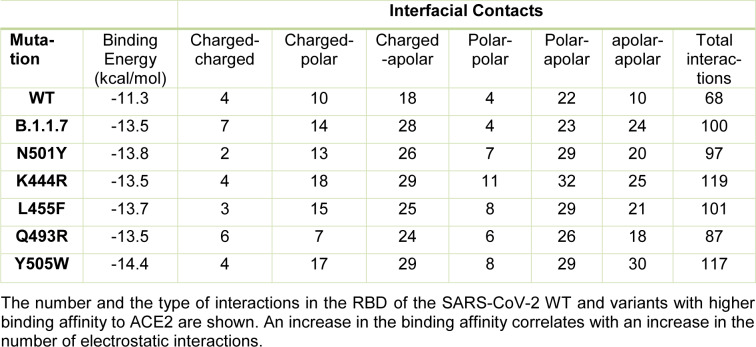
The binding characteristics between the SARS-CoV-2 spike protein mutants and ACE2 receptor

**Table 4:**
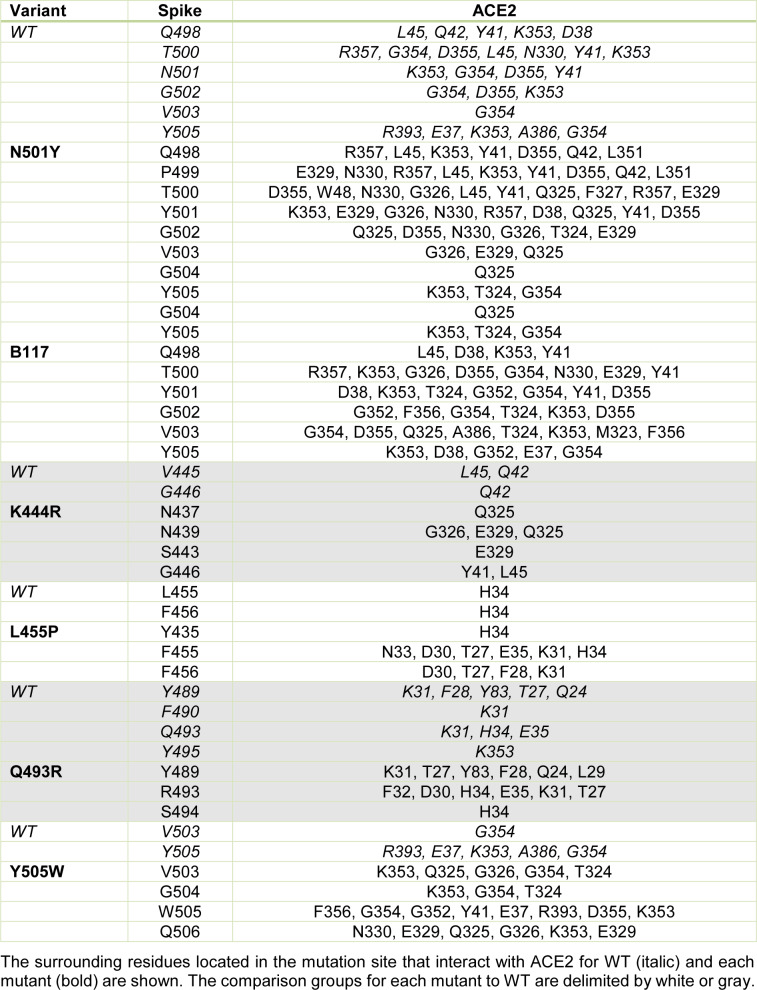
The close-up for the interaction between the mutated residues in the viral spike protein and the human ACE2

## References

[1] Arnold K, Bordoli L, Kopp J, Schwede T (2006). The SWISS-MODEL workspace: A web-based environment for protein structure homology modelling. Bioinformatics.

[2] Bobay LM, O'Donnell AC, Ochman H (2020). Recombination events are concentrated in the spike protein region of Betacoronaviruses. PLoS Genet.

[3] Cao H, Wang J, He L, Qi Y, Zhang JZ (2019). DeepDDG: Predicting the stability change of protein point mutations using neural networks. J Chem Inf Model.

[4] Chakraborty S (2021). Evolutionary and structural analysis elucidates mutations on SARS-CoV2 spike protein with altered human ACE2 binding affinity. Biochem Biophys Res Commun.

[5] Doud MB, Hensley SE, Bloom JD (2017). Complete mapping of viral escape from neutralizing antibodies. PLoS Pathog.

[6] Elbe S, Buckland-Merrett G (2017). Data, disease and diplomacy: GISAID's innovative contribution to global health. Glob Chall.

[7] Ewer KJ, Barrett JR, Belij-Rammerstorfer S, Sharpe H, Makinson R, Morter R (2021). T cell and antibody responses induced by a single dose of ChAdOx1 nCoV-19 (AZD1222) vaccine in a phase 1/2 clinical trial. Nat Med.

[8] Greaney AJ, Crawford KHD, Starr TN, Malone KD, Chu HY, Bloom JD (2020). Comprehensive mapping of mutations to the SARS-CoV-2 receptor-binding domain that affect recognition by polyclonal human serum antibodies. bioRxiv.

[9] Groves DC, Rowland-Jones SL, Angyal A (2021). The D614G mutations in the SARS-CoV-2 spike protein: Implications for viral infectivity, disease severity and vaccine design. Biochem Biophys Res Commun.

[10] Hoffmann M, Kleine-Weber H, Schroeder S, Kruger N, Herrler T, Erichsen S (2020). SARS-CoV-2 cell entry depends on ACE2 and TMPRSS2 and is blocked by a clinically proven protease inhibitor. Cell.

[11] Korber B, Fischer WM, Gnanakaran S, Yoon H, Theiler J, Abfalterer W (2020). Tracking changes in SARS-CoV-2 spike: Evidence that D614G increases infectivity of the COVID-19 virus. Cell.

[12] Kuriata A, Gierut AM, Oleniecki T, Ciemny MP, Kolinski A, Kurcinski M (2018). CABS-flex 2.0: A web server for fast simulations of flexibility of protein structures. Nucleic Acids Res.

[13] Laskowski RA, MacArthur MW, Moss DS, Thornton JM (1993). Procheck - a program to check the stereochemical quality of protein structures. J Appl Crystallogr.

[14] Leung K, Shum MH, Leung GM, Lam TT, Wu JT (2021). Early transmissibility assessment of the N501Y mutant strains of SARS-CoV-2 in the United Kingdom, October to November 2020. Euro Surveill.

[15] Lip KM, Shen S, Yang X, Keng CT, Zhang A, Oh HL (2006). Monoclonal antibodies targeting the HR2 domain and the region immediately upstream of the HR2 of the S protein neutralize in vitro infection of severe acute respiratory syndrome coronavirus. J Virol.

[16] Mathavan S, Kumar S (2020). Evaluation of the effect of D614G, N501Y and S477N mutation in SARS-CoV-2 through computational approach. Preprints.

[17] Ortega JT, Serrano ML, Jastrzebska B (2020). Class A G protein-coupled receptor antagonist famotidine as a therapeutic alternative against SARS-CoV2: An in silico analysis. Biomolecules.

[18] Ortega JT, Serrano ML, Pujol FH, Rangel HR (2020). Role of changes in SARS-CoV-2 spike protein in the interaction with the human ACE2 receptor: An in silico analysis. EXCLI J.

[19] Ortega JT, Serrano ML, Suarez AI, Baptista J, Pujol FH, Cavallaro LV (2019). Antiviral activity of flavonoids present in aerial parts of Marcetia taxifolia against Hepatitis B virus, Poliovirus, and Herpes Simplex Virus in vitro. EXCLI J.

[20] Pachetti M, Marini B, Benedetti F, Giudici F, Mauro E, Storici P (2020). Emerging SARS-CoV-2 mutation hot spots include a novel RNA-dependent-RNA polymerase variant. J Transl Med.

[21] Phillips JC, Braun R, Wang W, Gumbart J, Tajkhorshid E, Villa E (2005). Scalable molecular dynamics with NAMD. J Comput Chem.

[22] Pierce BG, Wiehe K, Hwang H, Kim BH, Vreven T, Weng Z (2014). ZDOCK server: Interactive docking prediction of protein-protein complexes and symmetric multimers. Bioinformatics.

[23] Pires DE, Ascher DB, Blundell TL (2014). DUET: A server for predicting effects of mutations on protein stability using an integrated computational approach. Nucleic Acids Res.

[24] Rambaut A, Loman N, Pybus O, Barclay W, Barrett J, Carabelli A,. on behalf of COVID-19 Genomics Consortium UK (CoG-UK) (2020). Preliminary genomic characterisation of an emergent SARS-CoV-2 lineage in the UK defined by a novel set of spike mutations. https://virological.org/t/preliminary-genomic-characterisation-of-an-emergent-sars-cov-2-lineage-in-the-uk-defined-by-a-novel-set-of-spike-mutations/563.

[25] Ravichandran S, Coyle EM, Klenow L, Tang J, Grubbs G, Liu S (2020). Antibody signature induced by SARS-CoV-2 spike protein immunogens in rabbits. Sci Transl Med.

[26] Romano M, Ruggiero A, Squeglia F, Maga G, Berisio R (2020). A structural view of SARS-CoV-2 RNA replication machinery: RNA synthesis, proofreading and final capping. Cells.

[27] Sharma A, Tiwari S, Deb MK, Marty JL (2020). Severe acute respiratory syndrome coronavirus-2 (SARS-CoV-2): A global pandemic and treatment strategies. Int J Antimicrob Agents.

[28] Singer J, Gifford R, Cotten M, Robertson D (2020). CoV-GLUE: A Web application for tracking SARS-CoV-2 genomic variation. Preprints.

[29] Sironi M, Hasnain SE, Rosenthal B, Phan T, Luciani F, Shaw MA (2020). SARS-CoV-2 and COVID-19: A genetic, epidemiological, and evolutionary perspective. Infect Genet Evol.

[30] Tang JW, Toovey OTR, Harvey KN, Hui DDS (2021). Introduction of the South African SARS-CoV-2 variant 501Y.V2 into the UK. J Infect.

[31] Toovey OTR, Harvey KN, Bird PW, Tang JWW (2021). Introduction of Brazilian SARS-CoV-2 484K.V2 related variants into the UK. J Infect.

[32] van Zundert GCP, Rodrigues J, Trellet M, Schmitz C, Kastritis PL, Karaca E (2016). The HADDOCK2.2 Web server: User-friendly integrative modeling of biomolecular complexes. J Mol Biol.

[33] Vanommeslaeghe K, Hatcher E, Acharya C, Kundu S, Zhong S, Shim J (2010). CHARMM general force field: A force field for drug-like molecules compatible with the CHARMM all-atom additive biological force fields. J Comput Chem.

[34] Vega S, Kang LW, Velazquez-Campoy A, Kiso Y, Amzel LM, Freire E (2004). A structural and thermodynamic escape mechanism from a drug resistant mutation of the HIV-1 protease. Proteins.

[35] V'kovski P, Kratzel A, Steiner S, Stalder H, Thiel V (2021). Coronavirus biology and replication: Implications for SARS-CoV-2. Nat Rev Microbiol.

[36] Wang P, Nair MS, Liu L, Iketani S, Luo Y, Guo Y (2021). Antibody resistance of SARS-CoV-2 variants B.1.351 and B.1.1.7. bioRxiv.

[37] Wei X, Decker JM, Wang S, Hui H, Kappes JC, Wu X (2003). Antibody neutralization and escape by HIV-1. Nature.

[38] Weisblum Y, Schmidt F, Zhang F, DaSilva J, Poston D, Lorenzi JC (2020). Escape from neutralizing antibodies by SARS-CoV-2 spike protein variants. Elife.

[39] Wiederstein M, Sippl MJ (2007). ProSA-web: Interactive web service for the recognition of errors in three-dimensional structures of proteins. Nucleic Acids Res.

[40] Xie X, Zou J, Fontes-Garfias CR, Xia H, Swanson KA, Cutler M (2021). Neutralization of N501Y mutant SARS-CoV-2 by BNT162b2 vaccine-elicited sera. bioRxiv.

[41] Xue LC, Rodrigues JP, Kastritis PL, Bonvin AM, Vangone A (2016). PRODIGY: A web server for predicting the binding affinity of protein-protein complexes. Bioinformatics.

[42] Zhang J, Liang Y, Zhang Y (2011). Atomic-level protein structure refinement using fragment-guided molecular dynamics conformation sampling. Structure.

