# Supplementary material for: Mutations in the SARS-CoV-2 spike protein modulate the virus affinity to the human ACE2 receptor, an in silico analysis
Source: EXCLI J. 2021 Mar 8;20:585–600. doi: 10.17179/excli2021-3471 (PMC8056063; doi:10.17179/excli2021-3471)
Supplement: Supplementary material [file EXCLI-20-585-s-001.pdf]

EXCLI Journal 2021;20:585-600 – ISSN 1611-2156
Received: February 02, 2021, accepted:February 22, 2021, published: March 08, 2021

Original article:

MUTATIONS IN THE SARS-COV-2 SPIKE PROTEIN MODULATE THE VIRUS AFFINITY TO THE HUMAN ACE2 RECEPTOR, AN *IN SILICO* ANALYSIS

Joseph Thomas Ortega[a], Flor Helene Pujol[b], Beata Jastrzebska[a]*, Hector R. Rangel[b]*

[a] Department of Pharmacology, Cleveland Center for Membrane and Structural Biology, School of Medicine, Case Western Reserve University, Cleveland, OH, USA
[b] Laboratorio de Virología Molecular, Centro de Microbiología y Biología Celular, Instituto Venezolano de Investigaciones Científicas, Apdo 20632, Caracas 1020A, Venezuela

* **Corresponding authors:** Hector R. Rangel, Ph.D., Laboratorio de Virología Molecular, Centro de Microbiología y Biología Celular, Instituto Venezolano de Investigaciones Científicas, Caracas, Venezuela; Phone: 58-412-7075300.
E-mail: hrangel2006@gmail.com
Beata Jastrzebska, Ph.D., Department of Pharmacology, Cleveland Center for Membrane and Structural Biology, School of Medicine, Case Western Reserve University, 10900 Euclid Ave, Cleveland, OH 44106-4965, USA; Phone: 216-368-4631;
Fax: 216-368-1300; E-mail: bxj27@case.edu

http://dx.doi.org/10.17179/excli2021-3471

This is an Open Access article distributed under the terms of the Creative Commons Attribution License (http://creativecommons.org/licenses/by/4.0/).

EXCLI Journal 2021;20:585-600 – ISSN 1611-2156
Received: February 02, 2021, accepted:February 22, 2021, published: March 08, 2021

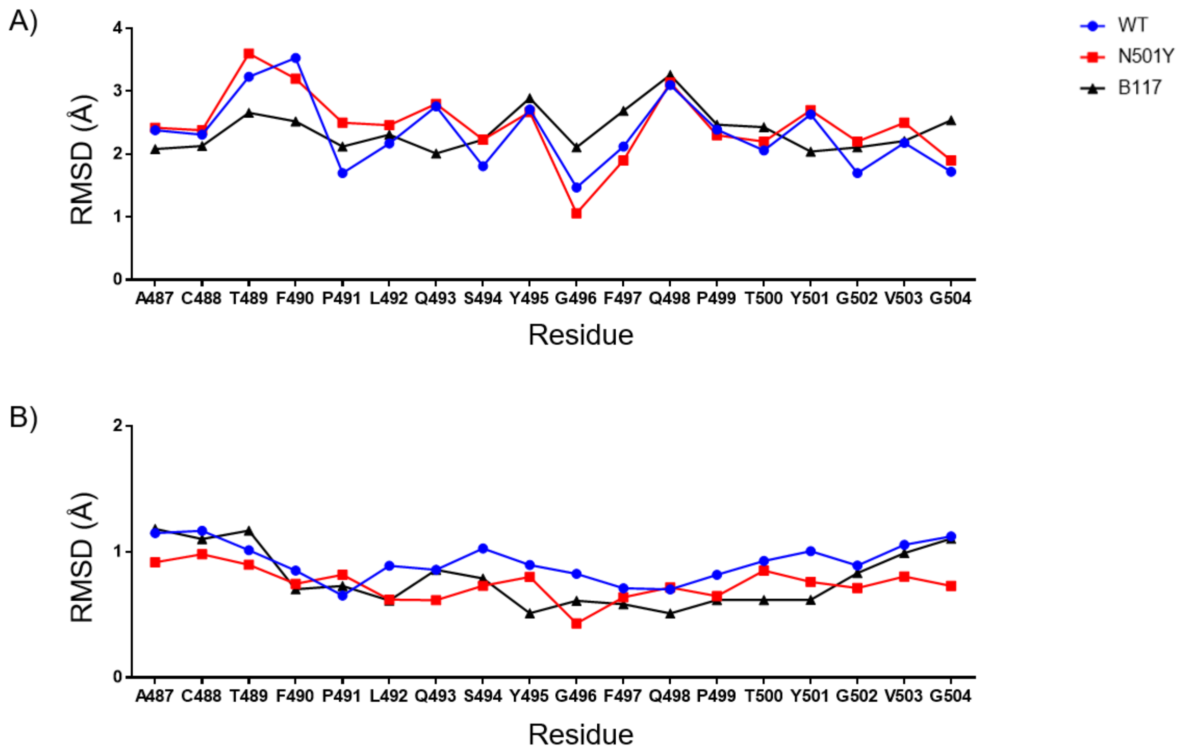

Supplementary Figure 1: The root-mean-square-deviation (RMSD) of the protein Cα atoms with the respect to the initial structure for the evaluated spike protein-ACE2 complex was obtained by molecular docking. **A**): The analysis performed with the VegaZZ software and **B**): The analysis performed with the CABS-Flex software.
